# Erratum: Breakthrough infections with the omicron and delta variants of SARS-CoV-2 result in similar re-activation of vaccine-induced immunity

**DOI:** 10.3389/fimmu.2023.1249100

**Published:** 2023-07-12

**Authors:** Frontiers Production Office

**Affiliations:** Frontiers Media SA, Lausanne, Switzerland

**Keywords:** vaccine, SARS-CoV-2, human, antibody, Breakthrough infection, cellular immunity

Due to a production error, the third author, Beathe Kiland Granerud, was not marked with equal contribution.

The publisher apologizes for this mistake.

The original version of this article has been updated.

