# Supplementary material for: Breakthrough infections with the omicron and delta variants of SARS-CoV-2 result in similar re-activation of vaccine-induced immunity
Source: Front Immunol. 2022 Sep 8;13:964525. doi: 10.3389/fimmu.2022.964525 (PMC9493489; doi:10.3389/fimmu.2022.964525)
Supplement: Supplementary file 1 [file DataSheet_1.pdf]

SUPPLEMENTAL MATERIAL:

Supplemental Table 1: The Excel file contains worksheets with source data for Figs 2-4 in context of vaccine status, type of infection and age. Separate sheets contain raw data for biochemical analysis and markers of inflammation and results from *in vitro* neutralization assays for SARS-CoV-2.

Supplemental Table 2. Clinical biochemistry at admission (T1) and 1-week follow-up (T2) in COVID-19 patients

		Omicron, n=52		Delta, n=18		
		Median (25th,75th)	Outside ref limit	Median (25th,75th)	Outside ref limit	Ref. limits
Hemoglobin, g/dL	T1	14.5 (13.5, 15.1)	2.0 %	14.0 (13.1, 14.5)	5.6 %	W: 11.7-15.3; M: 13.4-17.0
	T2	13.5 (12.7, 14.3)	14.6 %	13.6 (13.3, 13.8)	14.3 %	
Platelet counts, x10^9/L	T1	240 (205, 280)	4.0 %	234 (201, 307)	5.6 %	145-390
	T2	277 (236, 323)	5.0 %	297 (215, 324)	7.1 %	
WBC, x10^9/L	T1	5.2 (4.3, 5.9)	2.0 %	5.3 (4.5, 7.0)	0.0 %	3.5-10
	T2	5.7 (4.9, 6.6)	4.9 %	6.1 (5.4, 6.7)	0.0 %	
Neutrophils, x10^9/L	T1	2.6 (2.1, 3.2)	2.0 %	2.8 (2.3, 3.8)	5.6 %	1.5-7.3
	T2	3.3 (2.6, 4.0)	2.4 %	3.7 (3.0, 4.2)	0.0 %	
Lymphocytes, x10^9/L	T1	1.9 (1.6, 2.4)	5.9 %	1.9 (1.5, 2.4)	5.6 %	1.1-3-3
	T2	1.8 (1.5, 2.1)	4.9 %	1.8 (1.5, 1.9)	0.0 %	
Monocytes, x10^9/L	T1	0.4 (0.3, 0.5)	2.0 %	0.5 (0.4, 0.5)	5.6 %	0.2-0.8
	T2	0.4 (0.4, 0.5)	0.0 %	0.5 (0.4, 0.6)	0.0 %	
AST, U/L	T1	23 (20, 28)	5.8 %	26 (20, 29)	5.6 %	W: 15-35; M: 15-45
	T2	23 (19, 29)	7.1 %	20 (17, 25)	0.0 %	
ALT, U/L	T1	24 (18, 38)	0.0 %	23 (18, 31)	0.0 %	W: 10-45; M: 10-70
	T2	25 (17, 38)	4.8 %	19 (16, 23)	7.1 %	
Creatinine, µmol/L	T1	63 (59, 73)	0.0 %	66 (58, 76)	0.0 %	W: 45-90; M: 60-105
	T2	65 (61, 75)	0.0 %	69 (60, 79)	0.0 %	
eGFR, mL/min/1.73m^2	T1	113 (103, 119)	0.0 %	108 (100, 114)	0.0 %	>60
	T2	113 (102, 118)	0.0 %	104 (89, 111)	0.0 %	
Ferritin, µg/L	T1	119 (66, 221)	7.7 %	159 (72, 201)	5.6 %	W: 10-170; M: 30-400
	T2	103 (65, 191)	4.8 %	93 (48, 235)	21.4 %	
Fibrinogen, g/L	T1	3.3 (3.1, 4.1)	26.8 %	3.6 (3.2, 4.1)	27.8 %	1.9-4.0
	T2	2.7 (2.4, 3.2)	4.8 %	2.9 (2.2, 3.5)	0.0 %	
LD, U/L	T1	172 (160, 186)	9.6 %	169 (158, 178)	0.0 %	105-205
	T2	172 (158, 192)	11.9 %	161 (146, 178)	0.0 %	
ALP, U/L	T1	66 (51, 80)	1.9 %	64 (55, 79)	5.6 %	35-105
	T2	60 (50, 75)	2.4 %	63 (49, 69)	0.0 %	
CRP, mg/L	T1	1.8 (0.7, 4.9)	28.8 %	1.9 (1.0, 4.4)	27.8 %	<4
	T2	0.5 (0.5, 0.9)	4.8 %	0.6 (0.5, 1.1)	14.3 %	
Creatin kinase, U/L	T1	76 (60, 102)	1.9 %	72 (51, 104)	0.0 %	W: 35-210;

	T2	85 (67, 111)	2.4 %	70 (59, 93)	0.0 %	M: 50-400
D-dimer, mg/L FEU	T1	0.23 (0.18, 0.32)	7.3 %	0.23 (0.19, 0.31)	5.6 %	<50 yr: <0.50; >50 yr <age/100
	T2	0.19 (0.18, 0.32)	11.9 %	0.22 (0.18, 0.42)	14.3 %	
Troponin T, ng/L	T1	4 (4, 4)	1.9 %	4 (4, 4)	11.1 %	<14
	T2	4 (4, 5)	0.0 %	4 (4, 4)	7.1 %	
NT-proBNP	T1	49 (49, 49)	0.0 %	49 (49, 63)	0.0 %	W <50 yr: <170; >50 yr <300
	T2	49 (49, 62)	0.0 %	56 (49, 80)	0.0 %	M <50 yr: <85; >50 yr <250
IgG, g/L	T1	11.0 (10.0, 12.3)	3.8 %	10.8 (9.7, 12.4)	11.1 %	W: 6.9-15.7; M: 6.1-14-9
	T2	10.6 (9.3, 11.5)	0.0 %	11.0 (9.3, 12.1)	7.1 %	
IgA, g/L	T1	1.9 (1.65, 2.45)	7.7 %	2.8 (2.0, 3.1)*	16.7 %	0.7-3.7
	T2	1.8 (1.5, 2.2)	2.4 %	2.4 (1.9, 2.9)*	7.1 %	
IgM, g/L	T1	1.2 (0.89, 1.6)	7.7 %	1.35 (0.82, 1.7)	0.0 %	0.4-2.1
	T2	1.1 (0.83, 1.6)	2.4 %	1.05 (0.76, 1.6)	0.0 %	

*p<0.05 vs. omicron. WBC, white blood count; AST, aspartate aminotransferase; ALT, alanine aminotransferase; eGFR, estimated glomerular filtration rate; LD, lactate dehydrogenase; ALP, alkaline phosphatase; CRP, C-reactive protein; NT-proBNP, N-terminal pro-brain natriuretic peptide; Ig, immunoglobulin.

Supplemental Table 3: Markers of immune activation and inflammation in COVID-19 patients and healthy controls (HC) at admission (T1) and follow-up (T2).

		HC, n=13	Omicron, n=52	Delta, n=18	p-value
sCD14 µg/mL	T1	0.71 (0.44,0.82)	1.08 (0.95,1.21)***	1.09 (0.95,1.32)***	<0.001
	T2		0.73 (0.6,0.84)	0.63 (0.59,0.74)	0.12
sCD163 ng/mL	T1	325 (301,422)	540 (446,645)***	555 (489,726)***	<0.001
	T2		390 (327,510)†	340 (268,369)	0.035
MPO Ng/mL	T1	7.34 (5.76,11.07)	13.3 (7.3,17.16)	14.02 (10.51,17.91)	0.054
	T2		8.27 (6,10.88)	8.71 (5.3,14.12)	0.93
sCD25 pg/mL	T1	563 (459,603)	623 (508,875)	537 (430,708)	0.16
	T2		552 (411,730)	651 (456,705)	0.71
sTIM3 ng/mL	T1	5.17 (4.56,6.88)	6.03 (5.11,7.13)	6.12 (5.24,6.87)	0.46
	T2		5.21 (4.79,6.53)	5.57 (4.71,6.88)	0.89
LBP µg/mL	T1	6.48 (5.52,8.16)	8.88 (7.36,11.23)**	8.9 (7.15,10.59)**	0.010
	T2		7.48 (5.96,8.84)	8.73 (6.88,10.4)	0.072
TCC AU/mL	T1	0.87 (0.77,1.2)	0.94 (0.72,1.17)	0.91 (0.81,1.53)	0.76
	T2		0.87 (0.67,1.09)	0.94 (0.82,1.03)	0.45
IP10 Pg/mL	T1	80 (51,92)	113 (76,281)**	97 (71,162)*	0.030
	T2		72 (45,93)	69 (50,102)	0.89
vWF AU	T1	90.7 (87.9,97.2)	72.1 (58.9,88.9)**	79.7 (63.7,93.8)*	0.009
	T2		100.7 (86.3,112.9)	102.5 (92.2,108.8)	0.29
PTX3 Ng/mL	T1	1.26 (1,1.92)	2.29 (1.63,4.3)**	1.9 (1.52,3.51)*	0.009
	T2		1.76 (1.22,2.27)	2.39 (1.86,2.86)**	0.016
GDF15 Pg/mL	T1	201 (186,252)	322 (248,442)***	272 (244,365)*	0.001
	T2		304 (216,416)**	300 (228,377)*	0.010

YKL40 Ng/mL	T1	31.2 (28.3,40.9)	35.1 (28.7,40.4)	32.4 (30.2,43.7)	0.82
	T2		37.7 (31.5,47.1)	35.6 (28.5,53.5)	0.27
MMP9 Ng/mL	T1	28.1 (18.4,46)	25 (20.4,32.8)	27.9 (20.8,43.7)	0.83
	T2		27.5 (21.6,36.9)	26.6 (17.4,41.8)	0.97
S100A12 Ng/mL	T1	1.94 (1.34,2.33)	2.45 (1.69,3.85)	2.16 (1.42,3.92)	0.24
	T2		2.16 (1.84,2.93)	1.99 (1.45,3.43)	0.24

P-value in right column is from the a priori Kruskal-Wallis analysis. *p<0.05, **p<0.01, ***p<0.001 vs. HC. s, soluble; MPO, myeloperoxidase; TIM3, T cell immunoglobulin and mucin-domain containing-3; LBP, lipopolysaccharide binding protein; TCC, terminal complement complex; IP10, Interferon gamma-induced protein 10; vWF, von Willebrand factor; PTX3, Pentraxin 3; GDF15, Growth Differentiation Factor 15; YKL-40, Chitinase 3-like 1; MMP9, Matrix metallopeptidase 9; S100A12, S100 calcium-binding protein A12.
